# Three-Dimensional Nano-Morphology of Carbon Nanotube/Epoxy Filled Poly(methyl methacrylate) Microcapsules

**DOI:** 10.3390/ma12091387

**Published:** 2019-04-29

**Authors:** M. Galip Icduygu, Meltem Asilturk, M. Akif Yalcinkaya, Youssef K. Hamidi, M. Cengiz Altan

**Affiliations:** 1 School of Aerospace and Mechanical Engineering, University of Oklahoma, Norman, OK 73072, USA; mehmet.icduygu@giresun.edu.tr (M.G.I.); mltasilturk@gmail.com (M.A.); akifyalcinkaya@ou.edu (M.A.Y.); altan@ou.edu (M.C.A.); 2 School of Civil Aviation, Giresun University, Giresun 28200, Turkey; 3 Material Science and Engineering Department, Akdeniz University, Antalya 07070, Turkey; 4 Mechanical Engineering Program, University of Houston–Clear Lake, Houston, TX 77058, USA

**Keywords:** microcapsule, poly(methyl methacrylate), epoxy, carbon nanotubes, laser scanning confocal microscopy

## Abstract

The three-dimensional nano-morphology of poly(methyl methacrylate; PMMA) microcapsules filled with carbon nanotubes (CNTs) and epoxy resin were investigated by various microscopy methods, including a novel, laser scanning confocal microscopy (LSCM) method. Initially, PMMA microcapsules containing various amounts of CNTs were synthesized by a solvent evaporation method. Scanning electron microscopy analysis showed that pore-free, smooth-surface microcapsules formed with various types of core-shell morphologies. The average size of CNT/epoxy/PMMA microcapsules was shown to decrease from ~52 μm to ~15 μm when mixing speed during synthesis increased from 300 rpm to 1000 rpm. In general, the presence of CNTs resulted in slightly larger microcapsules and higher variations in size. Moreover, three-dimensional scans obtained from confocal microscopy revealed that higher CNT content increased the occurrence and size of CNT aggregates inside the microcapsules. Entrapped submicron air bubbles were also observed inside most microcapsules, particularly within those with higher CNT content.

## 1. Introduction

Microencapsulation is a process by which droplets or small particles, often referred to as the core, are enclosed within a shell to produce micro-scale capsules [1,2,3]. Microencapsulation was first introduced in the 1950s to produce carbonless copy paper from gelatin and gum arabic, which has been used commercially for many decades [4]. Interest in the microencapsulation technology has developed beyond printing, such that microcapsules are now used in a large number of applications, ranging from cosmetics and food additives to chemicals [5,6] and pharmaceuticals [7,8]. Thus, several microencapsulation techniques have been developed to effectively encapsulate a spectrum of material in gas, liquid, and solid phases. Depending on (a) the material to be encapsulated, (b) the method used to prepare the microcapsules, and (c) the desired application, shell materials are selected from a variety of synthetic or natural polymers. The resulting microcapsules exhibit various sizes (10–1000 μm in diameter) and shapes (ranging from simple spherical to irregular shells) [5,6,7,8,9,10].

In order to develop novel, versatile applications of these core-shell microcapsules, their synthesis and characterization have attracted extensive research efforts. Furthermore, incorporating nano-sized fillers into the microcapsules has gained momentum during recent years [11,12,13,14,15,16,17,18,19]. Among these nano-sized fillers, carbon nanotubes (CNTs) have attracted tremendous attention due to their exceptional intrinsic properties [20,21,22,23]. Adding well-dispersed CNTs to polymers such as epoxy yields improvements in mechanical, electrical, and thermal properties [20,21,22,23]. In a recent comparative study for instance, Zakaria et al. [23] reported a higher improvement in tensile and flexural strength for CNT/epoxy nanocomposites compared to epoxy filled with graphene nanoparticles. This article reports on the preparation and characterization of poly(methyl methacrylate; PMMA) microcapsules containing epoxy and CNTs. The aim is to develop a comprehensive understanding of the formation of microcapsules containing epoxy and CNTs. Once achieved, microcapsules with controlled characteristics such as size, shape, and CNT content and dispersion can be produced, and subsequently used for different potential applications. These applications range from damage sensing and electrochemical sensors [11,12,13], to electrical conductivity recovery [12] and self-healing of composites [14,15,16,17,18,19]. For instance, Caruso et al. [11] synthesized microcapsules containing CNTs using an in situ emulsification polymerization of urea–formaldehyde for damage sensing. The authors prepared microcapsules containing a suspension of CNTs in organic solvents by in situ polymerization, and studied their conductive behavior when ruptured. The intent was to use these CNT-filled microcapsules to restore electrical conductivity of a material that is lost upon mechanical damage [11]. Similarly, Odom et al. [12] studied autonomic restoration of electrical conductivity using polymer-stabilized CNTs and/or graphene microcapsules [12]. Delivering encapsulated conductive materials to damage sites in an autonomic fashion using core-shell microcapsules exhibits tremendous opportunity for restoring conductivity, and thus extend the lifetime of such devices [11,12,13]. Self-healing of polymer composites is another application that attracted significant attention. Several studies [24,25,26,27] reported remarkable potential of self-healing microcapsules for providing structural composites with increased life-span, yielding significant repair savings in terms of time and overhead costs. In these self-healing applications, crack growth within the composite matrix ruptures embedded microcapsules releasing both the resin and catalyst. Their localized mixing and subsequent curing recovers the properties at the damaged area. A number of studies [14,15,16,17,18,19] investigated incorporating CNTs into self-healing microcapsules. Fereidoon et al. [14], for example, investigated the effect of CNTs on the morphology and thermal properties of self-healing poly(urea-formaldehyde; PUF) microcapsules. CNTs were found to modify the physical properties of the PUF shell walls of microcapsules filled with dicyclopentadiene.

Solvent encapsulation has been commonly-used for encapsulation of epoxy [27], where epoxy and shell-forming polymer are dissolved in an organic solvent and dispersed in a water phase. After evaporation of the solvent, microcapsules with hard shells, which encase epoxy droplets, are obtained. The shells can be formed from a variety of polymeric materials such as poly(urea-formaldehyde) [11,12,13,14], urea-formaldehyde [28], melamine-formaldehyde [29,30], polyetherimide [31], cellulose, and ethyl cellulose [32]. Other shell materials include poly(ethylene glycol)s, poly(methacrylate)s, poly(styrene)s, poly(lactide)s, and gelatin [27]. In recent studies, poly(methyl methacrylate) PMMA was chosen for epoxy encapsulation as the capsule shell, due to its superior stability, biocompatibility, and good chemical compatibility with epoxies [33,34,35].

In solvent microencapsulation, a number of process parameters substantially affect microcapsule formation, shape and size distribution, and surface morphology [16,36,37]. These parameters include the mixing speed, the microcapsule core/shell weight ratio, the surfactant type, the reaction temperature, the emulsifier concentration, and the solvent evaporation temperature. Recently, two critical parameters have been identified for obtaining crack-free, smooth-surface microcapsules of PMMA capsules containing poly ether amine [36]. One of these parameters is the microcapsule core/shell weight ratio. Li and co-workers [36] observed that fractured capsules start to appear when the core/shell ratio exceeds 6/1. The second parameter is the evaporation temperature of the solvent. The authors also reported the formation of a porous capsule surface during the encapsulation process when the evaporation temperature was raised above 40 °C. Besides surface morphology, the average size and size distribution of PMMA microcapsules were found to correlate with the mixing speed used in the microencapsulation process [36]. A size distribution between 50 µm and 300 µm was obtained using mixing speeds ranging from 200 rpm to 500 rpm [36]. A similar trend was observed when poly(urea-formaldehyde) was used as the shell material [16,37].

One of the challenges of the solvent evaporation method is the agglomeration of microcapsules after evaporation in the absence of an appropriate surfactant. Therefore, the use of surfactants is necessary during the microencapsulation stage to ensure sufficient separation of microcapsules. However, the surfactant concentration can crucially affect the size distribution of the microcapsules [34,35]. For instance, higher surfactant concentrations yield smaller microcapsules and a narrower particle size distribution, whereas lower surfactant concentrations result in larger microcapsules and a wider size distribution [38,39]. Furthermore, the type of surfactant significantly affects the microcapsule structure. When using PMMA, sodium dodecyl sulfate (SDS), poly(vinyl alcohol), and cetyl trimethyl ammonium bromide as emulsifiers in the microencapsulation of various liquids, the size of the liquid droplets formed in emulsion were observed to be between 1.2 µm and 26.5 µm [35]. Poly(vinyl alcohol) yielded larger droplets with a broad size distribution, while PMMA and the smaller ionic surfactants formed smaller droplets with a lower polydispersity. This is directly related to the oil–water interfacial tension, which is lowered by a much greater extent when ionic surfactants are employed. Additionally, the emulsifier type not only affects the droplet size, but also significantly alters microcapsule morphology [35]. For instance, when SDS was used as a surfactant for hexadecane-PMMA microcapsule preparation, an “acorn” type morphology was obtained [35].

Different co-solvents can be used to control droplet size during emulsion. Using acetone and DCM, for example, as co-solvents for the preparation of PMMA microcapsules reduced both the average droplet size and its variation [35]. In a sense, the oil/water interfacial tension of the core material liquid exhibited a determining effect on droplet morphology, and “acorn”-like particles form at lower interfacial tensions between the aqueous phase and the core medium [35]. In fact, the core material characteristics play a critical role in the equilibrium morphology of microcapsules [35,36,40,41]. Depending on the chemical nature of organic phase such as polarity, hydrophobicity, ability to dissolve in the solvent used in encapsulation process, morphologies such as core/shell, occluded, acorn, or heteroaggregate can be formed [41]. The morphologies of interest for a microcapsule with a liquid core and a polymeric shell, i.e., core/shell and occluded, are known to form under particular conditions on the interfacial tensions of the dissolved phases as well as the organic solvent [42]. Furthermore, the organic phase viscosity is critical in obtaining isolated microcapsules [36]. During the evaporation process, droplets tend to come together and form larger, millimeter size droplets, and this tendency increases with increasing viscosity [36]. Using a low viscosity epoxy core, a low molecular weight PMMA, larger amounts of volatile solvent, and/or a higher evaporation temperature are reported to help avoid this phenomenon [35,36].

The extent of the sensing ability, electrical conductivity, or self-healing recovery may be improved if CNTs are added into the core material during the microencapsulation process. However, adequate dispersion is necessary to achieve the desired application improvement since CNTs often entangle together, form clusters, and micro-scale aggregates due to strong Van der Waals interactions between individual nanotubes. For instance, using CNT/epoxy dispersions in self-healing applications can improve the mechanical properties of both the healing agent at the damaged area, as well as the polymeric shell used in encapsulation process. In addition, a CNT-reinforced shell may lower the detrimental effect of voids formed after the rupture of microcapsules, and thus help improve retention of the mechanical properties in the self-healed material. Hence, microcapsules containing dispersed CNTs could have substantial potential for higher healing efficiency. Furthermore, the dispersion state of CNTs within the microcapsules would define the quality of the conductivity restoration in electronic devices.

A few studies [15,16,17,18,19,43] successfully produced microcapsules containing suspensions of CNTs. Yet, the state of CNT dispersion within the core material of the microcapsules has not been reported in the literature. Such information is necessary in order to develop a comprehensive understanding of the formation of microcapsules containing epoxy and CNTs. This understanding would allow the production of microcapsules, with controlled size, shape, and CNT content and dispersion, for different potential applications, including damage sensing, electrochemical sensors, electrical conductivity recovery, and self-healing of composites. This investigation focuses on characterizing the synergistic effects of CNT content and microencapsulation mixing speed on the microcapsule nano-morphology and the CNT dispersion. Consequently, we specifically investigated: (i) Producing PMMA microcapsules containing CNT/epoxy dispersion, (ii) probing submicron features of the formed microcapsules, and (iii) characterizing the three-dimensional CNT dispersion within the microcapsules using a novel non-destructive application of the laser scanning confocal microcopy (LSCM) method.

## 2. Materials and Methods 

### 2.1. Materials

EPON 828, i.e., difunctional bisphenol A/epichlorohydrin derived liquid epoxy resin (Miller Stephenson Chemical Co., Danbury, CT, USA), was used in this study as the core epoxy resin. In addition, Dichloromethane (DCM), PMMA (Mw ~120000), sodium dodecyl sulfate (SDS), and multi-walled carbon nanotubes (>90% carbon basis, D × L: 110–170 nm × 5–9 μm; Sigma Aldrich, St. Louis, MO, USA) were used in the microencapsulation process. These materials were used as received in order to avoid further costly processing that may limit the industrial use of the microcapsules. For instance, instead of CNT purification, sonication of CNT/epoxy mixtures was reported to be equally effective in alleviating the agglomeration or bonding challenges of CNT clusters [44,45].

### 2.2. Microcapsules Preparation

#### 2.2.1. Microencapsulation Parameters

In this study, the evaporation temperature was chosen as 40 °C and the core/shell ratio was optimized to get individual crack-free, smooth surface microcapsules as suggested by Li et al. [36]. The core/shell ratio was determined as 1:1 *w*/*w* to eliminate adhesion among epoxy/PMMA microcapsules and avoid agglomeration. Four different mixing speeds (i.e., 300, 500, 800, and 1000 rpm) and five CNT contents (0, 0.25, 0.5, 1, and 2 wt %) were used to prepare the microcapsules. A total of eleven microencapsulation configurations were used yielding different types of microcapsules. Table 1 lists the labels used for each type of microcapsule batches.

#### 2.2.2. Microencapsulation Procedure

**Figure 1:**
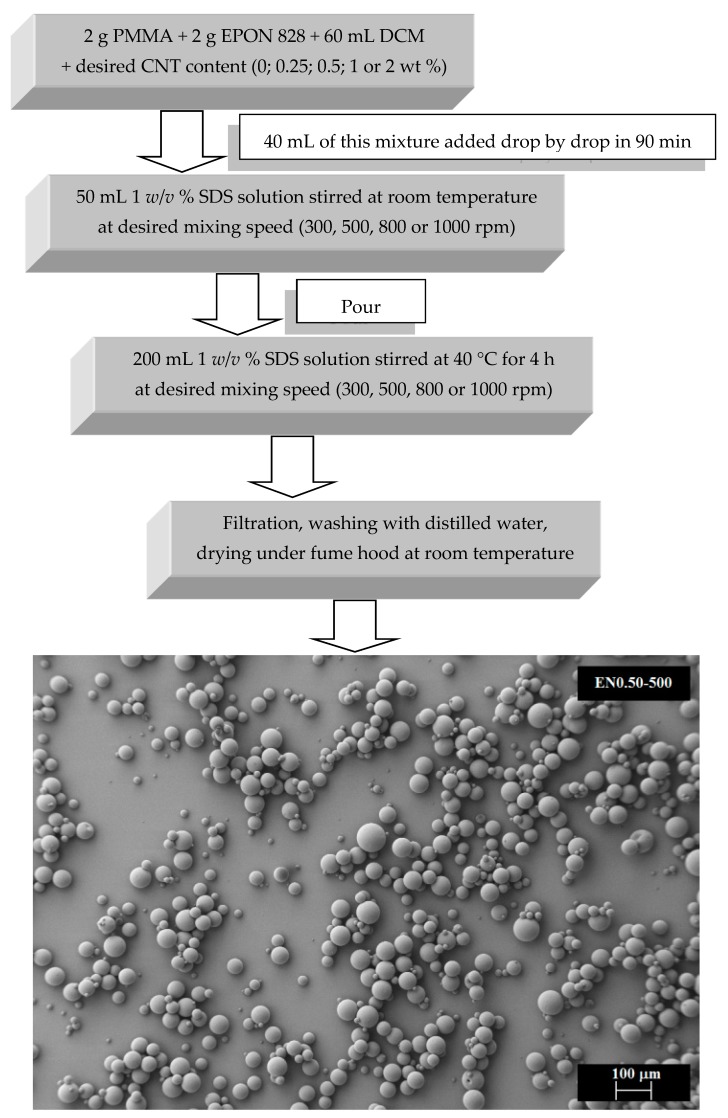
Preparation procedure of PMMA microcapsules containing epoxy/CNT mixtures.

The microcapsules were formed in an aqueous solution by solidification of PMMA at the resin-water interface following the preparation steps illustrated in Figure 1. First, 2 g of EPON 828 resin, 2 g of PMMA, and a predetermined amount of CNTs were mixed in 60 mL of DCM on a magnetic stirrer. DCM was selected since it acts as a solvent for both epoxy and PMMA. Once completely dissolved into DCM, the PMMA/epoxy/CNT mixture was sonicated to improve the CNT dispersion as suggested by Aktas et al. [45]. In fact, most of the favorable effects of the sonication, such as smaller cluster size and more uniform spatial distribution, were reported to occur early during a 5 s on/5 s off sonication scheme [45]. Therefore, the mixture was sonicated accordingly for 10 min on a Vibra-Cell, high intensity ultrasonic processor (Sonics & Materials, Newtown, CT, USA) at 55 W and 20 kHz.

Once the sonication was complete, 40 mL of this mixture was added drop by drop during 90 min at a particular mixing rate to 50 mL of a 1% *w*/*v* SDS solution to obtain the organic phase/water emulsion. After stabilization of emulsion, the mixture was added to a 200 mL 1% *w*/*v* SDS solution at the desired mixing rate, as suggested by Li et al. [36]. The mixture temperature was then raised to 40 °C to evaporate the solvent. After 4 h, the mixture was cooled to room temperature and filtered using Whatman Grade 93 filter paper. The solid product was washed with distilled water and dried at room temperature under a fume hood.

#### 2.2.3. Microcapsules Characterization

##### Fourier-Transform Infrared Characterization

Fourier-transform infrared (FTIR) spectra were obtained using a FTIR spectrometer (Bruker IFS 66/S Fourier Transform Infrared Instrument, Billerica, MA, USA) to identify the chemical composition of the microcapsules. Measurements were analyzed by signal averaging 16 scans at a resolution of 4 cm^−1^. Samples were prepared by mixing and grinding microcapsules with potassium bromide (KBr), and pellets were prepared under the press. The weight ratio of sample/potassium bromide in pellets was fixed as 1/80.

##### Thermal Stability

Thermogravimetric analysis (TGA) was used to verify the encapsulation of epoxy and CNTs, as well as to identify the maximum temperature at which the produced microcapsules could be used. TGA and derivative thermogravimetric (DTG) thermograms of the microcapsules were measured using a TG-DSC instrument (TA Instruments Q50, New Castle, DE, USA) at 10 °C min^−1^ heating rate from 30 °C to 700 °C in a nitrogen atmosphere (20 mL/min). Thermogravimetric analyses were performed for pure precursor and microcapsules (13 samples), and the weight of all samples were kept within a 25–30 mg range in an aluminum crucible.

##### Scanning Electron Microscopy (SEM)

The surface properties and shell morphologies of the microcapsules were investigated by examining ~500 images obtained using a ZEISS NEON 40 EsB scanning electron microscope (Zeiss, Oberkochen, Germany). Test samples were prepared by air-drying a drop of microcapsule suspension on a glass slide and then coated using a Hummer VI Triode Sputter Coater (Anatech USA, Hayward, CA, USA).

##### Laser Scanning Confocal Microscopy (LSCM)

LSCM is a unique light microscopy technique that enables nondestructive scanning and three-dimensional reconstruction of the microcapsules with a high resolution in all three imaging axes. Conventional microscopes are confined to cases where the light can be transmitted through the entire sample thickness. CNT clusters, when present in a sample, often obscure each other along the optical axis preventing in depth characterization. In order to bypass this limitation, LSCM uses the light in the reflected light path, while blocking any out-of-focus light. Additional desirable features of LSCM include its ability to characterize interior morphology and CNT dispersion of intact microcapsules with submicron resolution in all three dimensions.

Specifically, microcapsule size and morphology were characterized for each studied case using a Leica SP8 laser scanning confocal microscope (Leica Microsystems, Buffalo Grove, IL, USA) with 63× magnification in oil immersion. LSCM test samples were prepared by placing a drop of the microcapsule suspension on a glass slide and allowing it to air-dry. The samples were then immersed in oil. Each of the eleven investigated CNT content/mixing speed configurations listed in Table 1 was characterized using at least one LSCM sample group, among which three or more locations containing multiple microcapsules were scanned. The scans included optical sections with a thickness of 240 nm in the z-direction. The resolution of each optical section was 1024 pixels × 1024 pixels, which yielded a frame size of approximately 240 µm × 240 µm along the x- and y-directions. The captured LSCM scans were analyzed to accurately measure the size of each identified microcapsule by the image processing software, ImageJ (Fiji version). Depending on their size and concentration within each sample, 285 to 872 microcapsules were characterized for each of the eleven microcapsule types.

**Figure 2:**
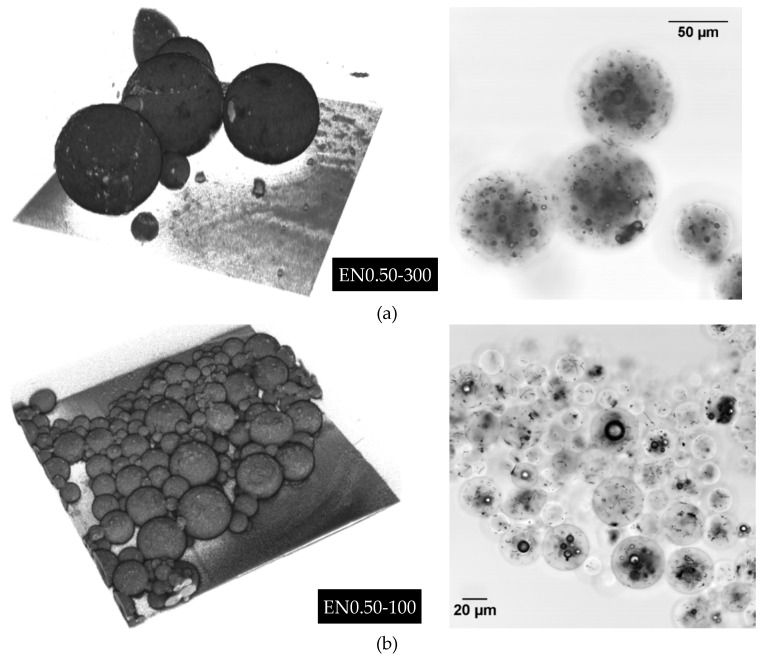

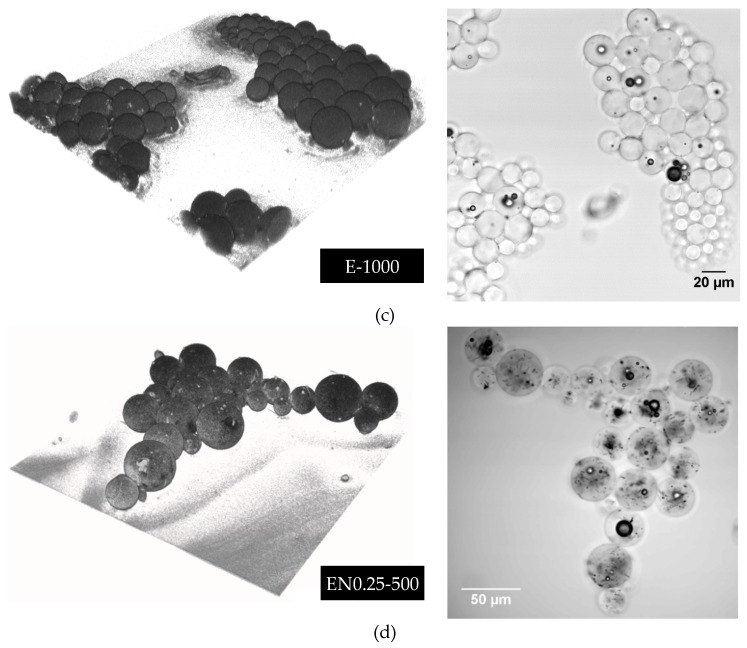
Representative images illustrating 3D reconstructions (left side) and mid-plane (right side) LSCM images of different microcapsules prepared at: (**a**) 300 rpm with 0.5 wt % CNTs, (**b**) 1000 rpm with 0.5 wt % CNTs, (**c**) 1000 rpm with no CNTs, and (**d**) 500 rpm with 0.25 wt % CNTs. Each 3D image is reconstructed from a stack of LSCM optical sections collected along the optical axis with a pixel size of 240 nm × 240 nm × 240 nm.

A stack of these optical sections was collected along the z axis (optical axis) for each location and then used to generate a 3D reconstruction of investigated volumes within each sample, with an approximate pixel size of 240 nm × 240 nm × 240 nm. Figure 2 shows 3D reconstructions (left side) and mid-plane (right side) images of different microcapsule types. With 3D reconstructions, it was possible to obtain additional information about the microcapsule morphology and shape that was not possible through SEM or optical microscopy. The aggregate nature of the spheres could yield inaccurate measurements from conventional 2D images, as smaller microcapsules might be concealed under the larger ones. Figure 2b,c depicts examples of such concealed microcapsules that would not have been captured by the conventional 2D imaging methods. In addition, optical sectioning enables non-destructive investigation of inner microcapsule structures. As the images showing the mid-plane through the thickness in Figure 2 (right side) demonstrate, the inner morphology features bubbles and CNT clusters of different sizes inside the microcapsules.

## 3. Results and Discussion

### 3.1. Chemical Composition of Microcapsules

**Figure 3:**
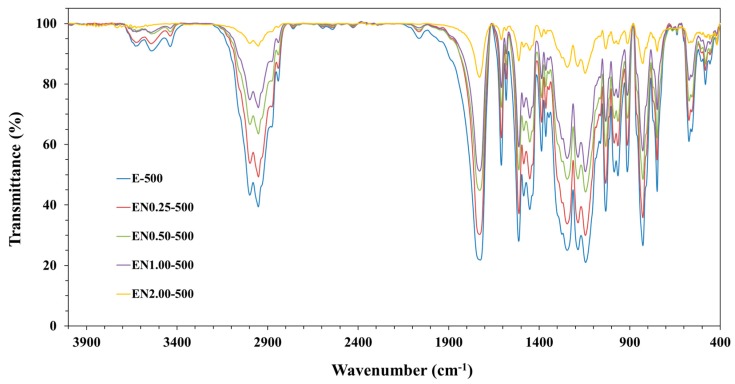
FTIR spectra of microcapsules prepared with different CNT contents.

In order to ensure that the fabricated PMMA microcapsules contain both epoxy and CNTs, FTIR analysis was conducted to characterize their chemical composition. Since the resin to polymer ratio was kept constant, the mixing speed was not expected to affect the chemical composition of the microcapsules. In addition, no chemical alteration was expected to occur in any constituents during the encapsulation process. As a result, the reactivity of the encapsulated epoxy was believed to remain identical to the neat epoxy. Furthermore, no difference in composition was observed when FTIR analysis was performed on microcapsules prepared at the different mixing speeds. Therefore, a single mixing speed was presented in Figure 3, depicting the FTIR spectra of microcapsules prepared with the different PMMA/epoxy/CNT mixtures at 500 rpm.

Figure 3 indicates that for all samples the absorption band of PMMA was between 1150 cm^−1^ and 1250 cm^−1^, which can be attributed to the C-O-C stretching vibrations. On the other hand, the band discerned at 1732 cm^−1^ substantiated the presence of acrylate carboxyl groups. Moreover, EPON 828 resin showed bands in the C=C phenyl ring stretching region between 1620 cm^−1^ and 1462 cm^−1^. The symmetric stretching band of the epoxy ring was also seen at 1250 cm^−1^, while the asymmetric ring stretching band of epoxy ring was located at 922 cm^−1^ and the out-of-plane bending band of p-substituted phenyl ring appeared at 825 cm^−1^. Therefore, the presence of EPON 828 resin and PMMA in the microcapsules was established in all samples prepared with different CNT contents.

Although CNTs were mixed within the PMMA/epoxy suspension, their presence inside the microcapsules needs to be corroborated as CNTs can be segregated and left out during the microencapsulation process. The clear decrease of the overall band intensity with increasing nanotube content in Figure 3 establishes that CNTs were effectively encapsulated into the PMMA microcapsules in higher ratios with increasing CNT contents.

### 3.2. Effect of Carbon Nanotube Content on Thermal Stability of Microcapsules

**Figure 4:**
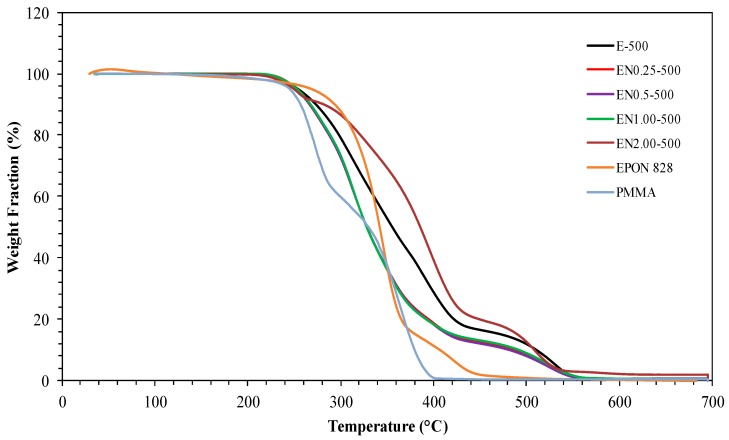
TGA thermographs of epoxy, PMMA, and PMMA microcapsules containing epoxy/CNT mixtures, under a nitrogen atmosphere at a heating rate of 10 °C min^−1^.

**Figure 5:**
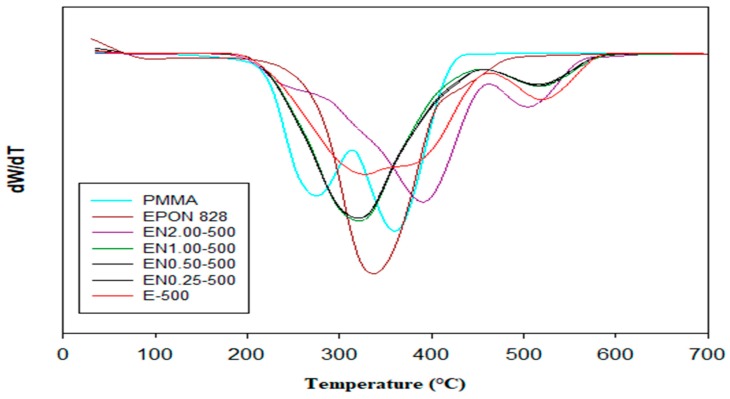
Derivative thermogravimetric (DTG) thermograms of epoxy, PMMA, and PMMA microcapsules containing epoxy/CNT mixtures, under a nitrogen atmosphere at a heating rate of 10 °C min^−1^.

Both Figure 4 and Figure 5 show TGA and DTG thermograms of EPON 828, PMMA, and PMMA microcapsules containing epoxy/CNT mixtures. Thermal decomposition for the neat PMMA occurred at between 180 °C and 400 °C, while, for EPON 828, thermal decomposition occurs between 250 °C and 600 °C. The contrasting thermal degradation behavior of the two polymers originates from the dissimilarities in their chemical compositions and end groups.

While the thermal decomposition of both epoxy resin and PMMA takes place in two stages, the decomposition of the microcapsules without CNT occurs in three stages. For these microcapsules, the first stage starts at around 240 °C; the second stage occurs between 350 °C and 400 °C; and the final stage takes place between 460 °C and 550 °C. The addition of CNTs was observed to increase the thermal stability of fabricated microcapsules. As depicted in Figure 4 and Figure 5, the thermal decomposition temperature of microcapsules increases with addition on CNTs. The PMMA was observed to decompose completely at 398 °C. The weight lost at this temperature for CNT containing microcapsules was found to be around 70%, while this value was around 88% for microcapsules with neat epoxy. This finding confirms the presence of epoxy in the microcapsules. An 81% weight loss was also observed for microcapsules containing 0.25 wt % and 1.00 wt % CNT. This indicates that the observed weight loss cannot be solely attributed to epoxy decomposition and CNT presence. Other factors, such as a varying amount of epoxy within the microcapsules, possibly caused by the entrapment of larger air bubbles, can affect the thermal decomposition behavior of microcapsules. The 68% weight loss observed at 398 °C for microcapsules containing 0.5 wt % CNT supports this finding. The weight loss of microcapsules with 2.00 wt % CNT content is around 59% at 398 °C, confirming that the presence of CNT increases thermal stability of microcapsules. 

In order to further confirm the thermal stability trend observed from TGA and DTG thermograms, an analysis of the heat-resistance index is performed on the investigated microcapsules. The heat-resistance index, T_HRI_, is a measurement of the ability of a material to resist a heat flow [46], and is given by Equation (1):

T_HRI_ = 0.49 × [T_5_ + 0.6 × (T_30_ − T_5_)], (1)

where T_5_ and T_30_ are the temperatures where 5% and 30% weight losses occur, respectively. Using the thermograms presented in Figure 4 and Figure 5, the heat-resistance index was calculated (Table 2) for epoxy, PMMA, and PMMA microcapsules containing epoxy/CNT mixtures.

Among the microcapsules constituents, PMMA exhibited the lowest heat-resistance index, followed by CNTs, then the neat epoxy. In fact, microcapsules containing neat epoxy exhibited a higher heat-resistance index than pure PMMA. The addition of CNTs, on the other hand, decreased this number by a considerable amount. Nonetheless, increasing CNT content was observed to increase the heat resistance-index. These results show that the fabricated PMMA microcapsules contained both epoxy and CNTs, corroborating TGA and FTIR findings. Furthermore, no thermal degradation was observed for any microcapsule type up to 200 °C, which makes them useful in applications performed below this temperature.

### 3.3. Effect of Mixing Speed and CNT Content on Microcapsule Size Distribution

**Figure 6:**
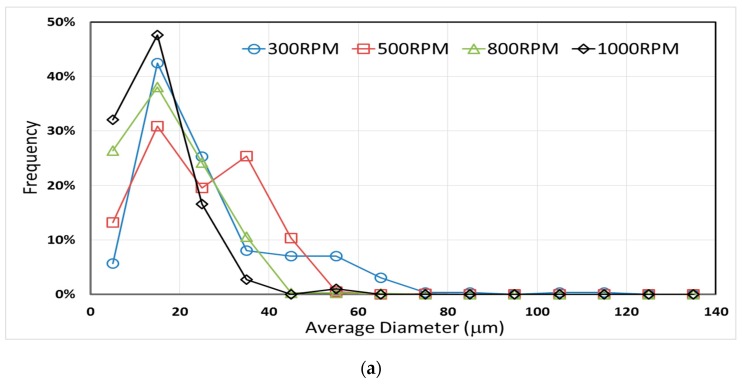

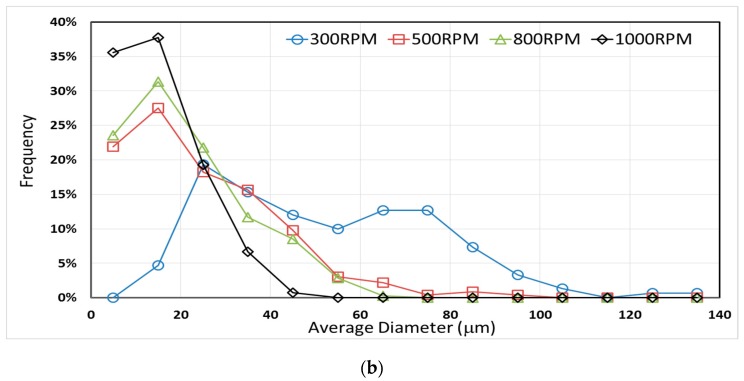
Size distribution of microcapsules prepared at different mixing rates (**a**) without CNTs, and (**b**) with 0.5 wt % CNTs.

The mixing speed used during microencapsulation is known to affect the size distribution of PMMA microcapsules containing epoxy resin [36,40]. However, the effect of CNT presence on the size distribution has not been investigated. Figure 6 shows both the effect of low carbon nanotube content (i.e., 0.5 wt %) and mixing speed of the PMMA/epoxy/CNT suspension on the size distribution of microcapsules.

In addition, Table 3 and Table 4 present the average and the maximum diameters of microcapsules prepared with different processing parameters. Using the 3D LSCM technique yields a more accurate determination of microcapsule size distribution compared to the commonly-used SEM characterization. In fact, LSCM allowed the identification and characterization of 15% to 25% additional smaller microcapsules that would be concealed under the larger ones in SEM images.

Further development of CNT-filled microcapsule applications would require efficient minimization of the property reduction induced by microcapsule presence within the material. This can be achieved by enhancing the interfacial adhesion between the microcapsules and the resin, and/or by reducing the size of the capsules to a level comparable to the diameter of fibers generally used in composites (i.e., 5 μm to 15 μm). Lowering the diameter of microcapsules would also reduce the volume of the contained substance in the composite, unless the amount of microcapsules is increased accordingly, which suggests an optimal amount and microcapsule size for efficient application in self-healing, conductivity recovery, or other applications [26,47]. Obtaining smaller capsules at 1000 rpm seems to have significant industrial applications, especially considering that almost 74% of the microcapsules formed were smaller than 20 μm and more than 92% were smaller than 30 μm. Additionally, these microcapsules can be sorted using sieve shakers, yielding uniform-sized capsules appropriate for particular applications.

Table 4 shows that both the average and maximum diameters decreased with increasing mixing speed. The average diameter shows a 70% decrease from 52.2 μm to 15.6 μm when the rpm was increased from 300 to 1000; while the maximum diameter shows a similar trend with a 71% decease. Nonetheless, once the CNTs were introduced to the PMMA/epoxy suspension, even at a concentration as low as 0.5 wt %, significant changes were observed on the size distribution of the prepared microcapsules at all mixing speeds. The average diameter of the microcapsules prepared at 300 rpm increased from 26.1 μm to 52.2 μm after CNTs were added. The almost two-fold increase was accompanied by a 25% increase in the maximum diameter. A milder trend was observed for the average diameter at higher mixing speeds as the increases were in the range of 1.7% to 6.1%. In addition, presence of CNT resulted in higher size variation as documented in Table 3 and Table 4, which show a two-fold increase in the 95% confidence intervals of average diameter of the 300 rpm, 500 rpm, and 800 rpm mixing speeds at 0.5 wt % CNTs. Figure 6b presents the size distributions of microcapsules formed at different mixing speeds with the presence of 0.5 wt % CNTs.

More insight into the effect of CNT presence on the variability of microcapsule size distributions can be obtained by comparing Figure 6a,b. At all mixing speeds, the presence of CNTs seems to promote the formation of both larger and smaller microcapsules, leading to a broader size distribution. For instance, the frequency of microcapsules formed at 300 rpm with a diameter larger than 50 μm expanded from 4.4% to 38.7% with the presence of 0.5 wt % CNTs. This increase in the frequency of microcapsules larger than 50 μm occurred at the expense of microcapsules smaller than 30 μm, as their frequency dwindled from 73.4% to 24.0%.

**Figure 7:**
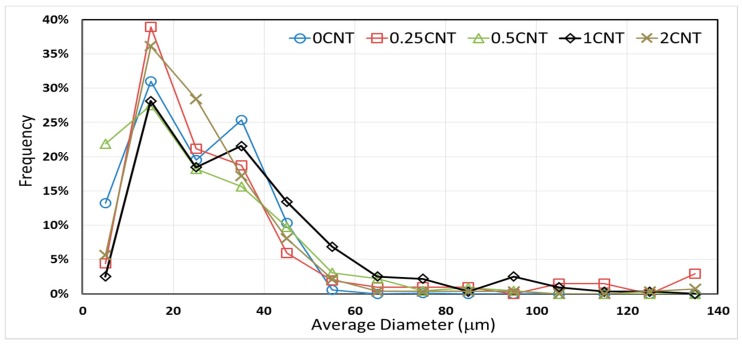
Size distribution of microcapsules prepared with different CNT contents at 500 rpm mixing speed.

The average and maximum diameters of microcapsules prepared at 500 rpm mixing speed with 0 wt %, 0.25 wt %, 0.5 wt %, 1 wt %, and 2 wt % CNT content are presented in Table 5. A clear trend was not observed for the average or the maximum diameters as the CNT content was increased. In addition, the variation in the microcapsule size was relatively high, as attested by the higher 95% confidence interval values. Figure 7 shows the effect of CNT content on microcapsule size distribution for a more detailed analysis.

None of the distributions showed a Gaussian trend, and a bi- or even multi-modal classifications might better describe the observed size distributions. The highest frequency peak at all CNT contents occurred at the 10 μm to 20 μm range. Other frequency peaks were observed at different ranges for different CNT contents. However, the general observed trend was that the introduction of CNTs promotes the formation of larger microcapsules. For instance, when the CNT content increased from 0 wt % to 1 wt %, the frequency of microcapsules larger than 50 μm rises from 0.7% to 15.9%. The significant CNT-induced increase in larger microcapsule frequency may be related to the increased viscosity of reacting mixture as well as the dispersion state of the CNTs within these mixtures. Increased viscosity enables the formation of larger emulsion droplets at any particular agitation rate. However, although droplet fragmentation is more delicate at higher viscosities, repetitive collisions between these larger microcapsules at different mixing speeds can break them down into smaller ones. Moreover, the presence of entangled nanotube clusters may facilitate the formation and retention of larger microcapsules.

### 3.4. Effect of Mixing Speed on Microcapsule Morphology

**Figure 8:**
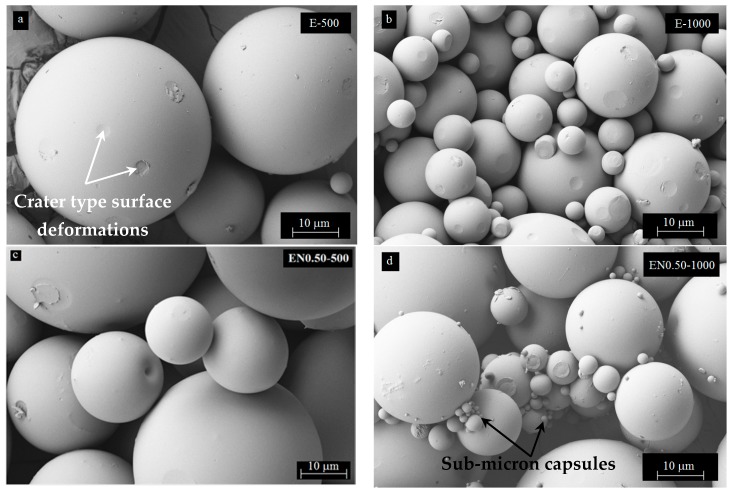
SEM micrographs showing the exterior surface of PMMA microcapsules containing epoxy/CNT mixtures prepared at: (**a**) 500 rpm with no CNTs, (**b**) 1000 rpm with no CNTs, (**c**) 500 rpm at 0.5 wt % CNTs, and (**d**) 1000 rpm at 0.5 wt % CNTs.

A detailed SEM characterization of the microcapsules would reveal the discernable effects of CNTs on the size variation and other morphological features of the microcapsules. As expected, larger microcapsules were observed at lower mixing speeds. On the other hand, the addition of CNTs promotes the formation of microcapsules with larger size variation. SEM images, depicted in Figure 8, show that the microencapsulation process produced spherical microcapsules with regular shapes and smooth surfaces. 

These findings disagree with those reported by Li et al. [40] for PMMA shell/epoxy core microcapsules prepared at 350 rpm mixing speed, for which rough microcapsule surfaces were observed under SEM. The difference in microcapsule surface may originate from the different core/shell ratio used in these studies. While a 1:1 *w*/*w* core/shell ratio was used in this study, a 4:1 *w*/*w* core/shell ratio was used by Li et al. [40], yielding a much thinner, and more deformable PMMA shell. Interestingly, Figure 8a–d, also exhibits crater type surface deformations, possibly caused by the collisions with neighboring spheres during mixing. This is a strong sign that PMMA forms a hard but somewhat ductile shell, which can be indented and plastically deformed without fracturing under external impacts. The collisions of the spheres seemed to have an accentuated effect on smaller microcapsules, as some loss their sphericity, which could be seen at the center of Figure 8b. Furthermore, roughness of the microcapsule surfaces was observed to increase with increasing mixing speed. This phenomenon can be related to both higher speed and rate of collisions between microcapsules during the encapsulation process.

**Figure 9:**
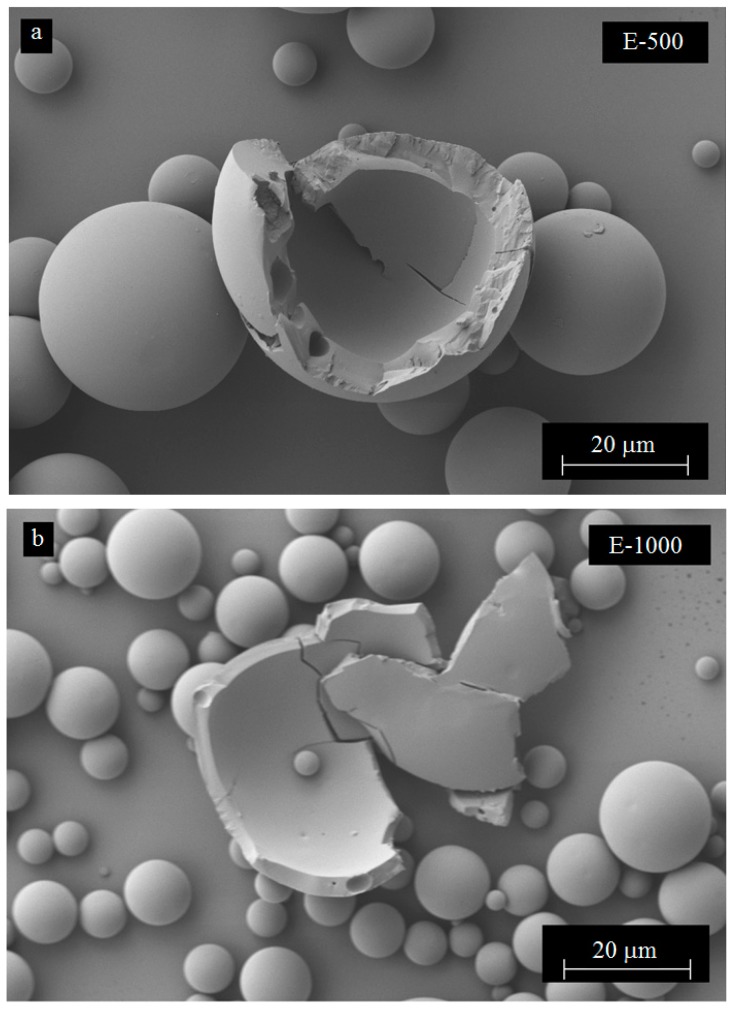

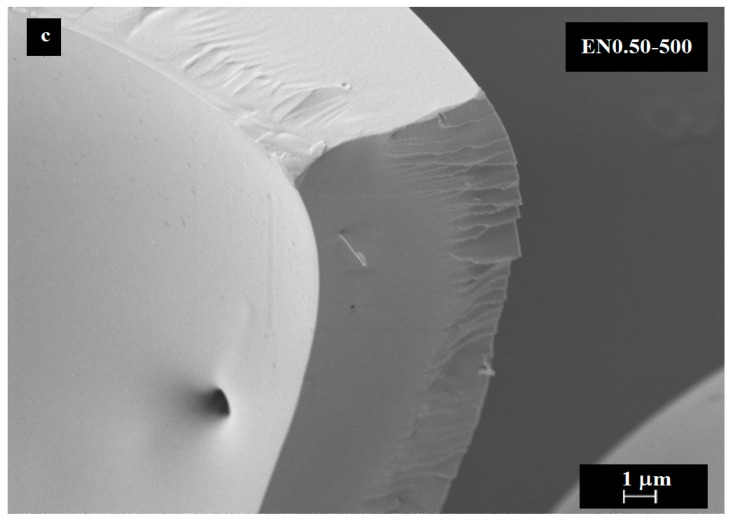
SEM micrographs showing interior surface and shell thickness of microcapsules containing epoxy/CNT mixtures prepared at: (**a**) 500 rpm with no CNTs, (**b**) 1000 rpm with no CNTs, and (**c**) 500 rpm with 0.5 wt % CNTs.

Additional SEM characterization of the fractured microcapsules shows that microcapsules with mono-nuclear core exhibited a uniform wall thickness as seen in Figure 9. The wall thickness varied between 1 microns and 3 microns and decreased with increasing mixing speed and microcapsule size. At 1000 rpm, submicron microcapsules started to appear and they formed clusters with bigger microcapsules by sticking onto their surfaces. SEM micrographs in Figure 9 mostly show smooth inner surface structures of the PMMA shells, even though some nanotubes appeared to be embedded within the shell as shown in Figure 9c. Pinhole-type small cavities were also visible on the interior surface of the microcapsules prepared without and with CNTs (Figure 9b,c, respectively). Pinhole-type formations may have formed as the escaping route for evaporating DCM during the earlier stages of microencapsulation. These perforations are closed from the outside during shell solidification after solvent evaporation.

Additional variations of the microcapsule shell structure were observed under SEM. For instance, some microcapsules were formed as solid spheres without any pinhole type formation, while others had spherical cavities unevenly distributed within the shell. These observed spherical cavities were enclosed within the shell and no pinholes were observed on the exterior or the interior surfaces of the microcapsules. In addition, analysis of almost 200 SEM images containing fractured microcapsules showed that the inner microcapsule and shell structures seemed to be independent from both CNT content and mixing speed.

### 3.5. Effect of Carbon Nanotube Content on Microcapsule Morphology

**Figure 10:**
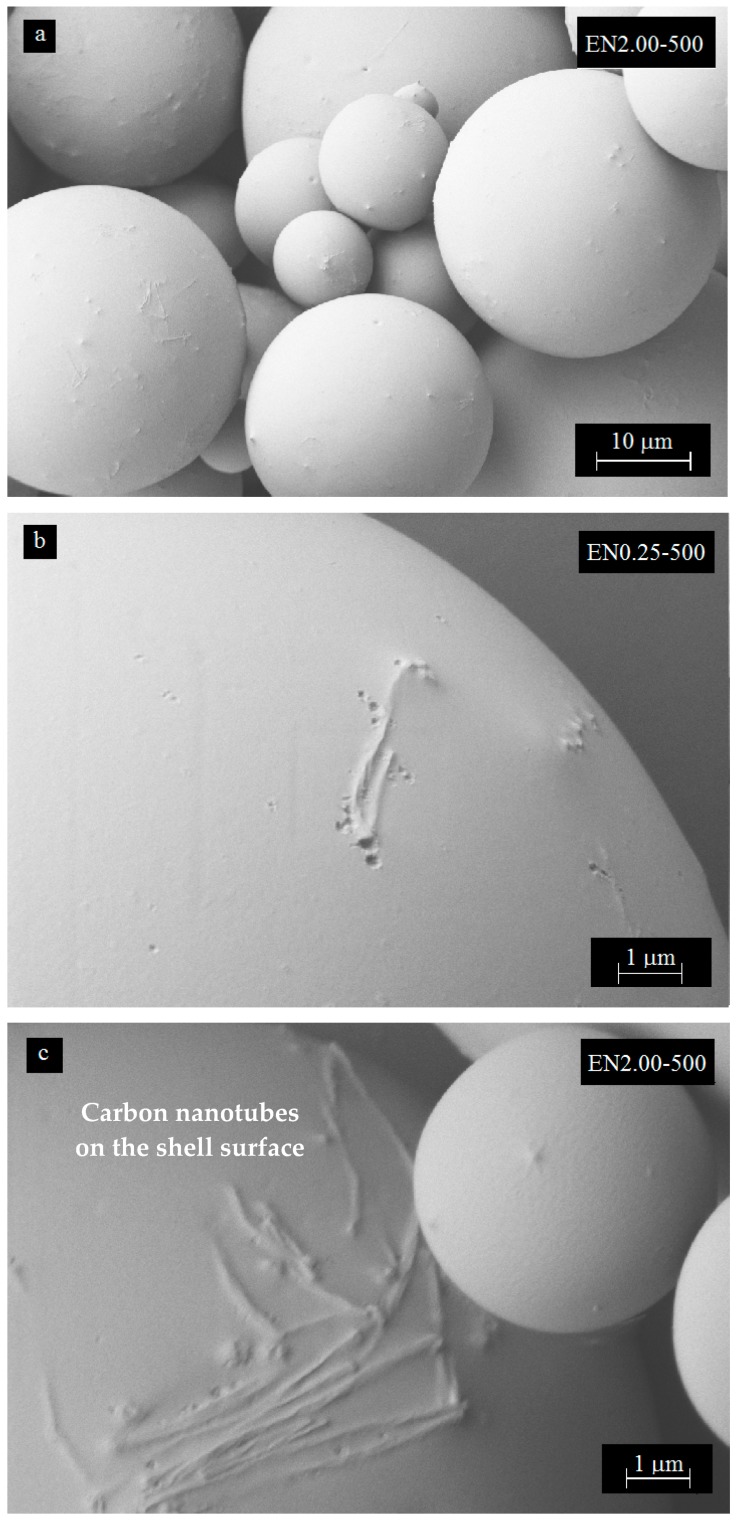
SEM micrographs showing CNT presence on the microcapsule exterior surface prepared with different CNT contents.

In addition to being embedded closer to the interior shell surface, CNTs were observed at the microcapsule surfaces as depicted in Figure 10a. Increasing the CNT content resulted in higher occurrences of such nanotube presence at the surface, which could be observed by comparing the surface of microcapsules prepared with 0.5 wt % CNT (Figure 8c) to 2 wt % CNT microcapsules (Figure 10a). Larger clusters on the shell surface were also observed at higher CNT contents as shown in Figure 10b,c. Some of these CNTs occurred in cluster formations, yielding nano-scale imperfections on the surface of microcapsules. The density of these clusters was low and the CNTs in the clusters were observed to conform to the spherical curvatures of the shell periphery.

Presence of CNTs in the PMMA microcapsule shell can provide reinforcement and increase its mechanical properties, which can be beneficial for CNT-filled microcapsule applications. First, the CNT-reinforced shells may fracture instead of bending inward or buckling, which may facilitate the release of the core liquid. Second, reinforced microcapsule shells can support the cavity that may possibly be formed after the core material is released, and thus may help maintain the structural integrity of the material.

**Figure 11:**
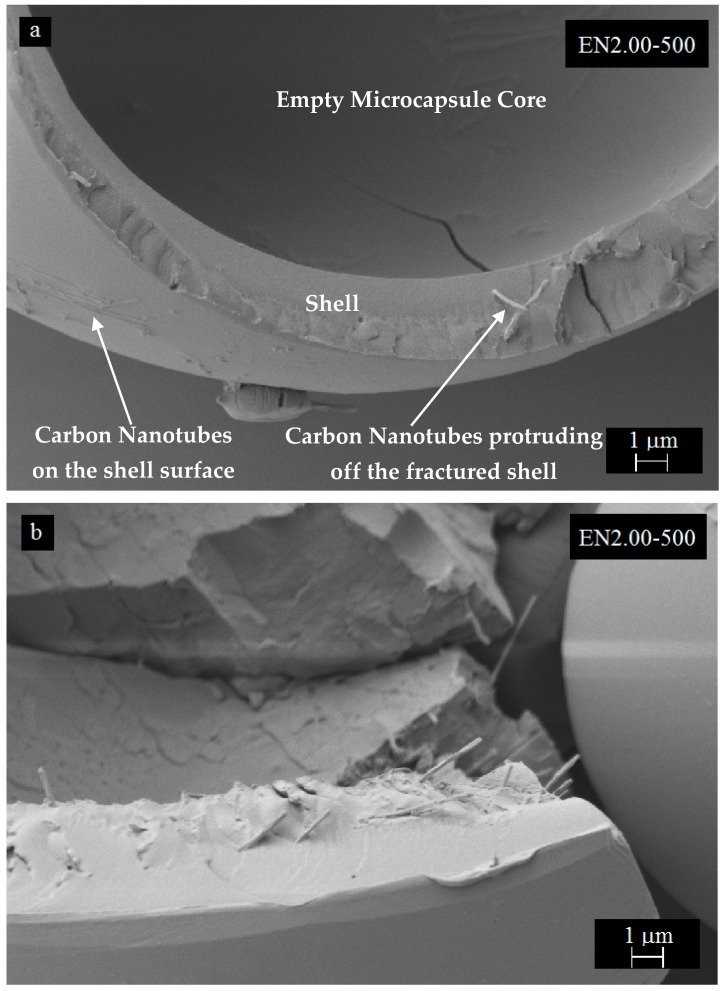
SEM micrographs of microcapsule formed with 2 wt % CNT: (**a**) Close view of the capsule interior surface and shell structure, and (**b**) CNT presence in the fractured shell.

A closer analysis of microcapsule surfaces indicated that CNTs were unevenly distributed on the surface as shown in Figure 10. In addition, the number of clusters was observed to increase with increasing CNT content. The surface roughness was also observed to be closely related to the amount of CNT used in the microencapsulation process. The increased surface roughness is a favorable feature for CNT-filled microcapsule applications, since it would enhance the interfacial area and the adhesion between the microcapsules and the matrix. In fractured microcapsules, CNTs were eventually observed protruding from the shell surface as shown in Figure 11a,b.

Moreover, CNTs exhibit an uneven distribution through-the-thickness of the PMMA shell, and they tend to form mostly circumferential clusters located within the shell thickness. Fracture surfaces of microcapsule shells also seemed to be influenced by the CNT content used during encapsulation. Repeated collisions cause mostly a clean fracture surface. However, the fracture characteristics of the shell changes with increasing CNT content, and cone shape fractures were observed in microcapsules with 2 wt % CNT content.

The findings for PMMA microcapsule morphology in this study contrast with those reported for poly(urea formaldehyde) microcapsules [15,16,18,19,43,48]. Microcapsules containing epoxy resin and nano-sized fillers in the literature are dominantly prepared using poly(urea-formaldehyde) as the encapsulating shell material [15,16,18,19,43,48]. Microcapsules obtained by suspension polymerization of urea-formaldehyde were reported to exhibit a spherical shape with rough surfaces [43]. In another study [18], it was observed that poly(urea-formaldehyde) microcapsules prepared with CNTs did not have spherical shapes. These conflicting observations may originate from the core/shell ratio used to produce each microcapsule type, as thinner poly(urea-formaldehyde) walls can be more prone to deviate from spherical shapes during mixing. Furthermore, no CNTs were observed on the poly(urea-formaldehyde) shell surface. Rather, CNTs were observed to form a cage-like structure inside the microcapsules [48]. CNTs seem to disperse relatively well in both PMMA and epoxy resin [45,49,50]. On the other hand, CNTs have been reported to require some form of functionalization in order to disperse into poly(urea-formaldehyde) [18,19]. The use of non-functionalized CNTs might explain the absence of CNTs from poly(urea-formaldehyde) microcapsule shells reported in the literature.

**Figure 12:**
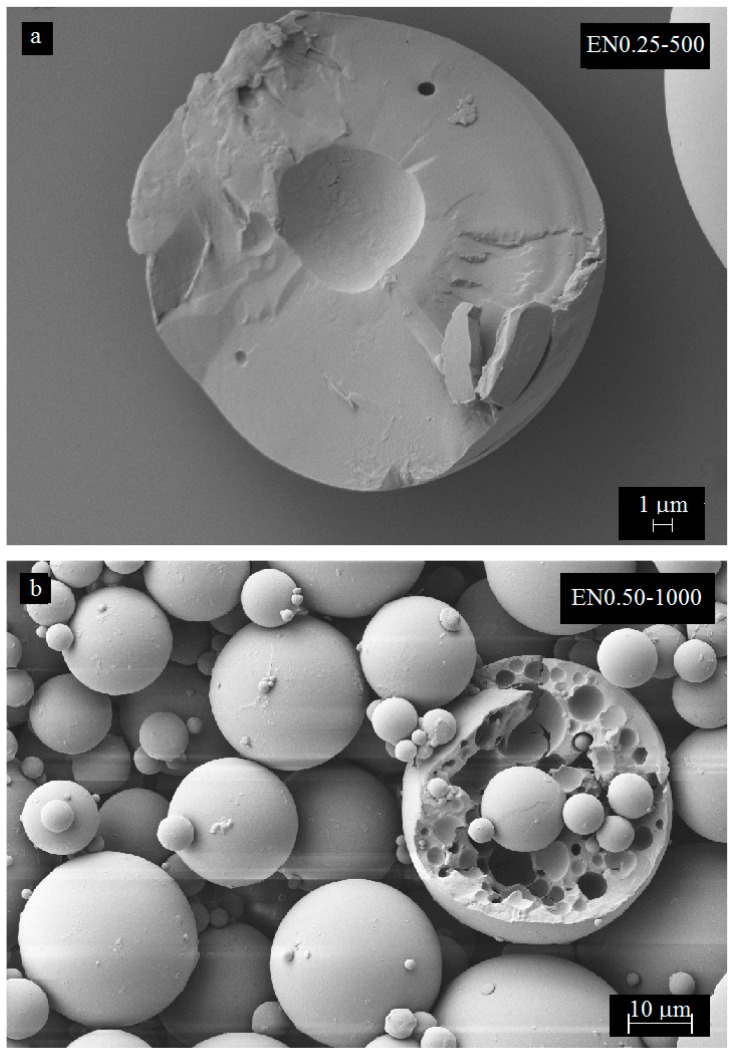

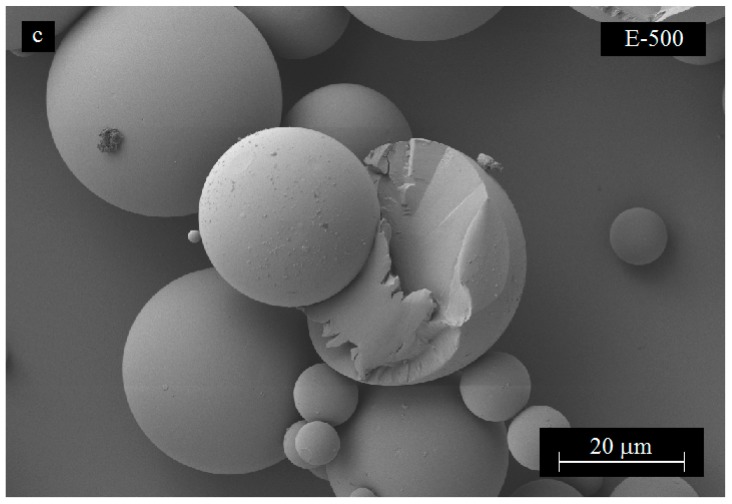
SEM micrographs showing additional shell and core morphological features observed on the prepared microcapsules.

Various other morphological features were also observed from the SEM images, including microcapsules with no core, multi-core, or small core. Microcapsules with a very small core (Figure 12a), or with a homogenous solid PMMA sphere (Figure 12c) were observed to form during the encapsulation process. In addition, microcapsules with sponge-like porous structure were observed as presented in Figure 12b. Occasionally, shell and core morphologies vary even when the same microencapsulation parameters are used. Further investigation of these inner morphological features would require using non-destructive techniques and three-dimensional scanning methods such as LSCM.

### 3.6. Three-Dimensional Morphology of CNT Dispersion in Microcapsules

**Figure 13:**
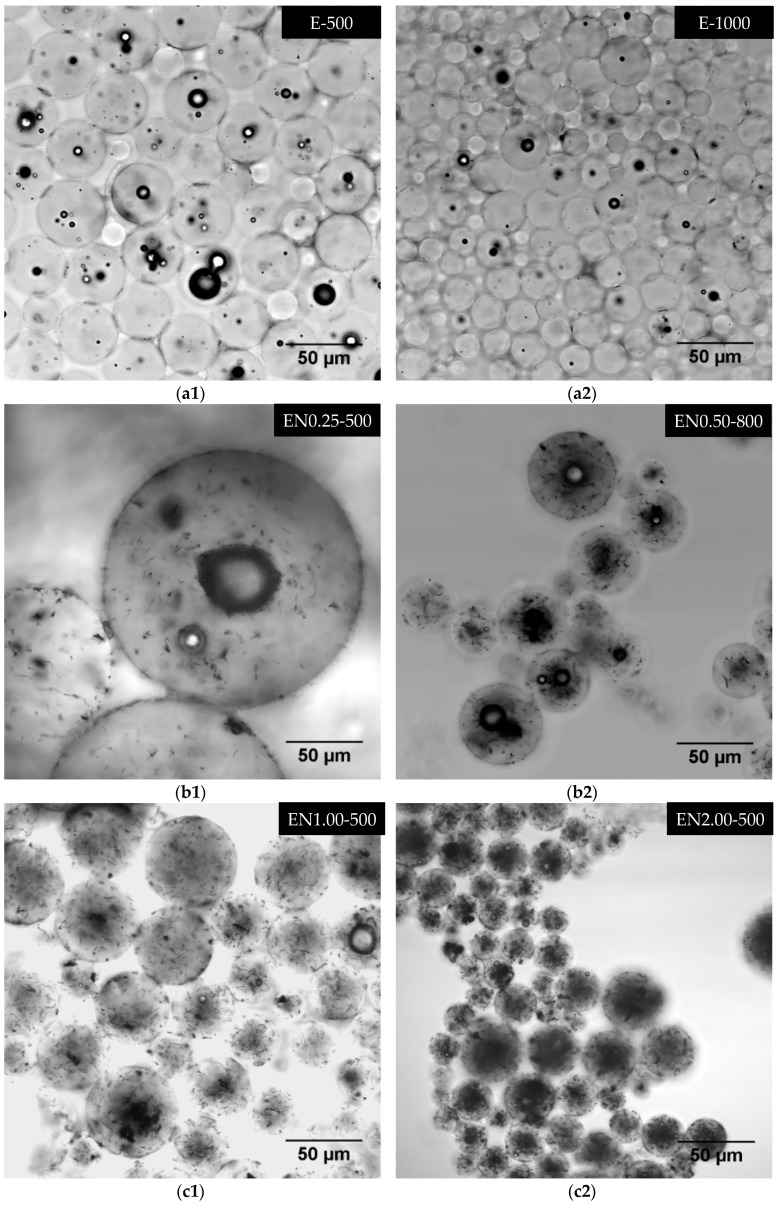

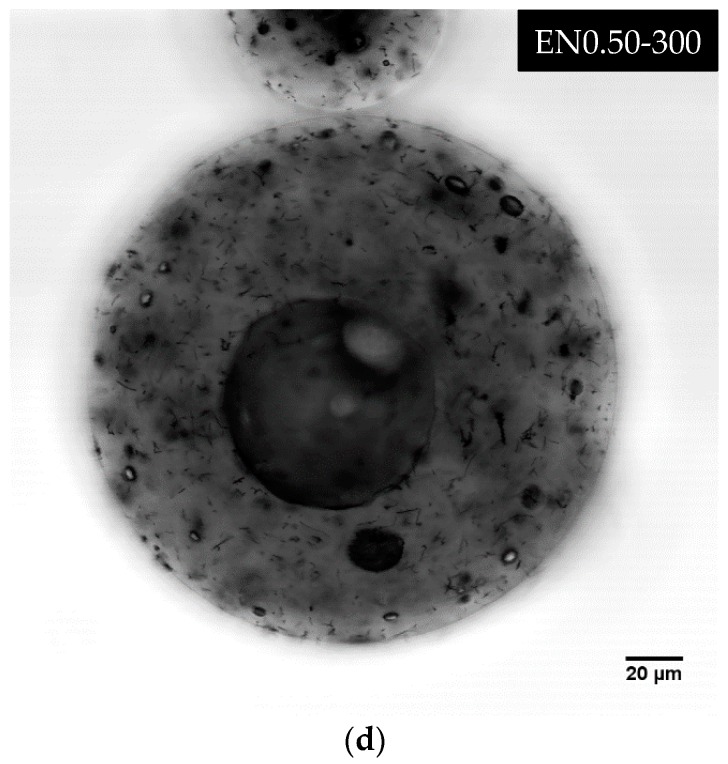
Laser scanning confocal microscopy (LSCM) micrographs showing morphological features observed within the microcapsules.

In this study, a novel application of the LSCM imaging was implemented to non-destructively probe submicron features of the microcapsules, and investigate three-dimensional CNT dispersion. Figure 13 depicts a variety of morphological features, including CNT dispersion and clustering encountered in microcapsules. A number of small air bubbles, or voids, were observed to be present in all types of microcapsules, regardless of the microencapsulation parameters and CNT content. Bubble occurrence and their size were observed to decrease with increasing mixing speed for microcapsules prepared without CNTs, as shown in Figure 13a1,a2.

The same trend was observed for microcapsules containing CNTs (Figure 13b). Much fewer and smaller bubbles were observed within the microcapsules prepared at 300 rpm without CNTs. Conversely, microcapsules prepared at 300 rpm with 0.5 wt % CNTs exhibited the highest occurrence of smaller voids (Figure 13d). Presence of CNTs seemed to promote the entrapment of air bubbles, as no bubble-free morphology was observed for microcapsules containing CNTs. Increasing CNT content, on the other hand, seemed to promote the formation of smaller voids within the same microcapsule, as seen in Figure 13b1,b2,c1, and c2. The highest occurrence of voids was observed at 2 wt % CNTs. Furthermore, larger void occurrence seemed to depend primarily on the microcapsule size.

Another important observation was that the presence of these small air bubbles was not exclusive to the epoxy microcapsule core, as such voids were also observed at the PMMA shell (Figure 13d). These air bubbles possibly form during emulsion at different mixing speeds, and their occurrence can be related to differences in the micro-flow characteristics, such as turbulence, viscosity, and local CNT content at the epoxy/PMMA interface. Presence of CNTs, especially entangled clusters, may locally increase the viscosity of the mixture, thus promoting the entrapment of additional and/or larger air bubbles during the microencapsulation process. These voids may also form during the evaporation of DCM, when the CNT-reinforced PMMA shell does not shrink to compensate for the lost solvent volume. Excessive amount of air entrapped within the microcapsules may alter its thermal stability, which might explain the slight deviation in 2 wt % CNT microcapsule thermograms (Figure 4 and Figure 5).

**Figure 14:**
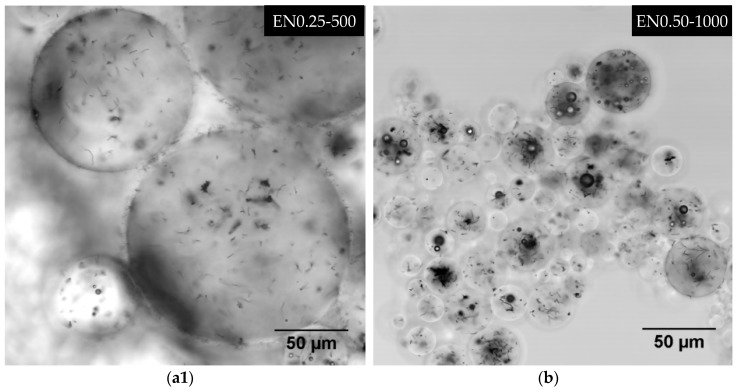

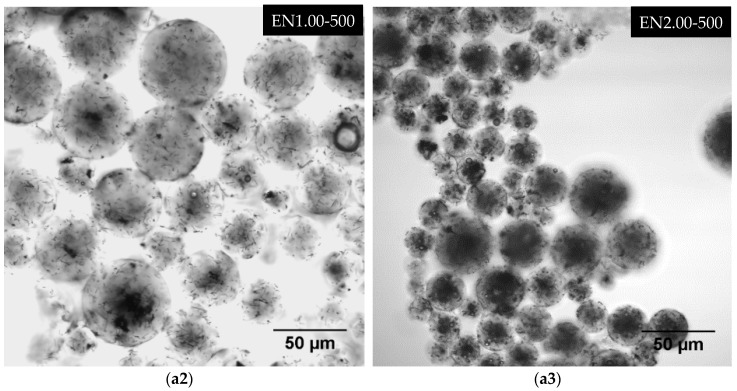
Laser scanning confocal microscopy (LSCM) micrographs showing CNT dispersion features observed within the microcapsules.

Figure 14 depicts different dispersion states observed within microcapsules prepared with different process parameters. Typically, CNTs do not agglomerate or form large clusters as seen in Figure 14a1. Seldom occurrence of entangled clusters with sizes ranging between 0.6 µm × 0.3 µm and 10 µm × 5 µm was observed at all CNT contents and all mixing speeds. Such CNT clusters could be seen around the center of the large microcapsule depicted in the right bottom corner of Figure 14a2. Present in both the epoxy core and the PMMA shell, these clusters were observed to have a random three-dimensional orientation, which indicates the formation of isotropic CNT/epoxy microcapsules. At low CNT content, most CNT clusters were observed in the range of 0.6 µm × 0.3 µm to 2 µm × 3 µm. As the CNT content increased, the density of these clusters augmented and a higher occurrence of larger clusters, i.e., in the range of 5 µm × 3 µm to 10 µm × 5 µm, was observed. This rise in cluster occurrence did not seem to affect the three-dimensional random orientation of CNTs. Therefore, no process-induced anisotropy was expected within the formed microcapsules at higher CNT contents. Figure 14a2,a3 show a higher concentration of these larger entangled clusters within smaller microcapsules at 1 wt % and 2 wt % CNT content, respectively.

As the mixing speed was increased, the uniformity of CNT dispersion from one microcapsule to another was observed to decrease. At 800 rpm, smaller microcapsules deprived of any CNTs were observed. A higher occurrence of these CNT-deprived microcapsules was observed at 1000 rpm. Concurrently, microcapsules containing higher concentrations of CNT were also observed as shown in Figure 14b.

## 4. Conclusions

PMMA microcapsules containing epoxy and various carbon nanotube (CNT) content were prepared using the solvent evaporation technique. Different process parameters were varied in order to develop a comprehensive understanding of the microencapsulation process of epoxy and CNTs. The results indicated that microcapsules can be produced with controlled characteristics such as size, shape, and CNT content and subsequently used for a wide variety of applications.

Microcapsules with hole- or rupture-free surfaces were formed by mixing speeds from 300 rpm to 1000 rpm at 40 °C evaporation temperature. FTIR spectra analysis corroborated that the produced microcapsules contained both PMMA and epoxy as well as CNTs. Thermal stability analysis of the microcapsules showed that the amount of encapsulated epoxy slightly varied, but remained mostly unaffected by the microencapsulation process parameters. Both the mixing speed and the CNT content played an important role in the overall morphology of microcapsules. Higher mixing speeds were found to yield smaller microcapsules. Increasing the mixing speed from 300 rpm to 1000 rpm caused a significant decrease in the average diameter of microcapsules from 26 μm to 14 μm at 0 wt % CNT and from 52 μm to 15 μm at 0.5 wt % CNT, respectively. SEM images showed that microcapsules exhibited smooth shell surfaces, with various types of core-shell morphologies. However, increasing both the mixing speed and CNT content yielded slightly higher surface roughness of the microcapsules as entangled CNTs clustered at the shell surface.

A novel application of the laser scanning confocal microscopy imaging enabled non-destructive, three-dimensional probing of interior morphological features of the microcapsules at the submicron scale, which could not be realized by conventional microscopy or SEM imaging methods. CNTs were observed in both PMMA shell and epoxy core, and higher CNT content was found to increase the occurrence and density of entangled CNT clusters inside the microcapsules, without affecting their three dimensional random orientation. Interestingly, air bubbles of different sizes were observed to be trapped inside the microcapsules. Presence of CNTs seemed to promote bubble formation, while higher mixing speed decreased bubble occurrence and size. Increasing CNT content was also found to augment the occurrence and the density of CNT entangled clusters. As the mixing speed was increased, the uniformity of CNT dispersion was observed to decrease.

## Figures and Tables

**Table 1:** Labels of microcapsule batches with different carbon nanotube (CNT) contents formed at different mixing speeds.

Sample Code	CNT Content (wt %)	Mixing Speed (rpm)
E-300	0	300
E-500	0	500
E-800	0	800
E-1000	0	1000
EN0.25-500	0.25	500
EN0.50-300	0.50	300
EN0.50-500	0.50	500
EN0.50-800	0.50	800
EN0.50-1000	0.50	1000
EN1.00-500	1.00	500
EN2.00-500	2.00	500

**Table 2:** Temperature values at 5% and 30% weight loss and heat resistance index of epoxy, PMMA, and PMMA microcapsules containing epoxy/CNT mixtures.

Sample Code	T_5_ (°C)	T_30_ (°C)	T_HRI_ (°C)
PMMA	245.37	279.32	130.21
EPON 828	269.38	327.82	149.17
E-500	254.87	317.36	143.26
EN0.25-500	252.71	304.15	138.95
EN0.50-500	252.86	303.89	138.90
EN1.00-500	253.69	304.88	139.36
EN2.00-500	249.80	348.63	151.46

**Table 3:** Effect of mixing speed on average and maximum microcapsule diameters at 0 CNT content.

Sample Code	Average Diameter (μm) ± 95% Confidence Interval	D_max_ (μm)
E-300	26.1 ± 1.9	119.4
E-500	24.2 ± 0.8	75.8
E-800	17.5 ± 0.7	62.5
E-1000	14.7 ± 0.9	57.1
E-300	26.1 ± 1.9	119.4

**Table 4:** Effect of mixing speed on average and maximum microcapsule diameters at 0.5 wt % CNT content.

Sample Code	Average Diameter (μm) ± 95% Confidence Interval	D_max_ (μm)
EN0.50-300	52.2 ± 4.0	149.3
EN0.50-500	24.6 ± 1.5	95.5
EN0.50-800	21.2 ± 1.3	69.6
EN0.50-1000	15.6 ± 1.0	42.8

**Table 5:** Effect of CNT content on average and maximum microcapsule diameters.

Sample Code	Average Diameter (μm) ± 95% Confidence Interval	D_max_ (μm)
E-500	24.2 ± 0.8	75.8
EN0.25-500	31.8 ± 4.4	215.3
EN0.50-500	24.6 ± 1.5	95.5
EN1.00-500	34.0 ± 2.0	127.0
EN2.00-500	26.0 ± 2.3	154.7
